# Common Peroneal Nerve Palsy Due to Giant Fabella After Total Knee Arthroplasty

**DOI:** 10.1111/os.12874

**Published:** 2021-02-23

**Authors:** Ke Shen, Pengcheng Bai, Ran Sun, Lei Liu, Fei Wang, Baicheng Chen, Xiaofeng Wang

**Affiliations:** ^1^ Department of Orthopaedic Surgery Third Hospital, Hebei Medical University Shijiazhuang China

**Keywords:** Common peroneal nerve, Fabella, Palsy, Total knee arthroplasty

## Abstract

**Background:**

Common peroneal nerve palsy (CPNP) is a rare but serious complication following primary total knee arthroplasty (TKA). The common peroneal nerve is one of the main molecules of the sciatic nerve. CPNP is a series of symptoms caused by common peroneal nerve injury due to paralysis and atrophy of the fibula and tibia muscles. The main clinical symptoms are: ankle joint unable to extend back, toe unable to extend back, foot droop, walking in a steppage gait, and foot dorsal skin sensation having decreased or disappeared. If treatment is not timely, severe cases may result in atrophy of the anterior tibia and lateral calf muscles. The risk factors for CPNP include mechanical stretching of the nerve, disruption of the blood supply to the nerve, and compression of the nerve. The CPNP should be treated in a timely manner and according to the cause. Its function should be restored as soon as possible to avoid serious adverse consequences. It has negative effects on patients’ life and physical and mental health. To our knowledge, this is the first study to describe CPNP due to a giant fabella after TKA.

**Case presentation:**

The present study reported on a 70‐year‐old female patient. The patient underwent a primary TKA of the right knee for osteoarthritis. Relevant examinations were conducted and the operation went smoothly. Three hours postoperation, a right partial CPNP was observed, with progressive aggravation over time. On palpation, there was a 2 × 2‐cm fixed hard mass in the posterolateral aspect of the right knee, with mild tenderness to deep palpation. Radiographs demonstrated that a giant fabella was located at the posterolateral condyle of the right femur. Fabellectomy and neurolysis of the common peroneal nerve were performed. The peroneal nerve palsy resolved gradually after the operation. At 8‐month follow up after fabellectomy and neurolysis, the function of the common peroneal nerve had fully recovered.

**Conclusions:**

The presence of giant feballa pressing on the common peroneal nerve should be considered when common peroneal nerve palsy occurs after TKA. Surgical exploration and release compression should be performed in a timely manner.

## Graphical abstract

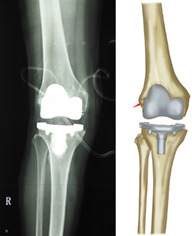

Postoperative X‐ray: There was a giant fabella after total knee arthroplasty at the posterior upper part of the external femoral condyle (Left). Schematic digram of the Graphical abstract image. The red arrow idcicates the giant fabella (Right).

## Introduction

A sesamoid bone is a small endophytic bone formed by ossification of tendons under high pressure. The purpose of the sesamoid bone is mainly to strengthen the tendons and avoid tendon wear during exercise and heavy physical labor. The patella is the largest sesamoid bone in the human body, and everyone has it. Other parts of the sesamoid bone vary from person to person; some people have it and some people do not. Sesamoid bones appear in other parts of the body, which is related to the normal life. They frequently grow at the base of the foot. Rounded or ovoid sesamoid bones located in the lateral head of the gastrocnemius muscle are referred to as faba bean bones or small sural bones because of the similar shape. It can change the pressure conduction, reduce the friction between the tendon and the bone surface, change the direction of muscle traction, increase the efficiency of muscle contraction, and stabilize the knee joint. The lateral sural bone is closely related to the common peroneal nerve, which can pass from the anterior and posterior lower part, to the surface and the anterior and upper part. Normal small sural bones do not cause symptoms, and the incidence of sural bones is higher in patients with clinical osteoarthritis. The larger sural bones often develop marginal sclerosis, forming an articular surface with the external condyle of the femur. A diseased or abnormal sural bone may cause symptoms such as pain or paralysis of the lateral knee.

Common peroneal nerve palsy (CPNP) after total knee arthroplasty (TKA) is a rare but potentially devastating complication, with a reported incidence ranging from 0.3% to 4% after primary TKA^1^, ^2^, ^3^, ^4^. Previous studies have reported risk factors for CPNP after TKA, including correction of valgus or flexion deformity during TKA^3^, ^5^, prolonged tourniquet time^6^, epidural anesthesia^5^, ^7^, higher body mass index (BMI)^1^, compression of the nerve due to a pseudotumor after TKA^8^, and previous spinal pathology^9^, ^10^, but the cause of peroneal nerve palsy may not be clearly identifiable in most cases. Fabellae are sesamoid bones that are visible at radiography in approximately 10–30% of the general population and are found bilaterally in most cases^11^. However, CPNP associated with a giant fabella after TKA have not been previously reported. A case of CPNP due to a giant fabella following a TKA is described.

We have the approval of the ethics committee of our institution, and we obtained permission from the patient to publish this data.

## Case Report

A 70‐year‐old woman was admitted with double knee osteoarthritis. The patient had no peripheral neuropathy. The BMI of the patient was 19 kg/m^2^. The range of motion (ROM) of the right knee was 10°–130°, and there was mild varus deformity (5°) of the right knee. We performed TKA of the right knee with the patient in the supine position under general anesthesia, using a medial parapatellar approach and a cemented cruciate retaining prosthesis (Waldemar Link, Hamburg, Germany), without patella resurfacing. During the operation, a tourniquet was placed at thigh level with a pressure of 230 mmHg. The tourniquet remained inflated for 55 min. The total operative time was 70 min. Before closing the wound, ropivacaine hydrochloride (10 mL, AstraZeneca AB, SE‐151 85 Sodertalje, Sweden) was injected around the knee for postoperative pain control. The estimated blood loss was 100 mL. Dressings were applied to protect the operative site, and the operative lower limb was wrapped by elastic bandages from foot to thigh. The operation went smoothly.

At 3 h postoperation, the patient was found to have significant motor weakness of the right ankle and great toe dorsiflexion (MRC grade 3/5) and toe dorsiflexion (MRC grade 4/5) and hypoesthesia over the superolateral aspect of the dorsum of the right foot, the first web‐space, and the inferolateral crural region. The clinical findings suggested an incomplete CPNP. Therefore, we immediately removed the elastic bandages, loosened the wound dressings, and positioned the knee at 20° flexion. Radiography of the right knee immediately after surgery demonstrated that the prostheses in the right knee were well cemented, and the components were well positioned. There was a fabella behind the posterior condyle of the femur prosthesis, but we paid no further attention to it at the time. The drainage tube was removed at 24 h after surgery. Physical examination revealed that the operative knee was slightly swollen, and there was no obvious effusion in the joint. However, the presentation of CPNP was slightly worse. The patient was given a physical therapy program involving full range of active and passive motion exercises of the involved ankle and toes. On the second day after the operation, the patient was found to have worse motor weakness of the right ankle and great toe dorsiflexion and toe dorsiflexion (MRC grade 2/5, 2/5, 3/5, respectively) and worse hypoesthesia over the superolateral aspect of the dorsum of the right foot, the first web‐space, and the inferolateral crural region. The patient showed signs of foot drop and was placed in an ankle–foot orthosis. The full range of active and passive motion exercises of the involved ankle and toes was continued.

On the third day after the operation, the muscle strength of the right ankle and great toe dorsiflexion and toe dorsiflexion fell to MRC grade 1/5 and 3/5, respectively. The symptoms of the CPNP in the patient deteriorated with time; therefore, we supposed that the nerve palsy was not caused by intraoperative injury nor due to compression of dressings. The common peroneal nerve was likely damaged by compression. Physical examination revealed that the operative knee was slightly swollen, there was no redness, and there was no obvious effusion in the joint. However, there was a 2 × 2‐cm hard lump that could be palpated in the lateral aspect of the popliteal fossa of the right knee, which was tender to deep palpation. A hard lump was found at the same site of the left knee but no pain. CT images obtained at 3 days post‐TKA identified a giant fabella over the posterolateral condyle site of the femur, located at the front of the common peroneal nerve. Postoperative radiography of the right knee demonstrated that there was a giant fabella behind the posterior condyle of the femur prosthesis. In view of the findings, we determined that it was the giant fabella which oppressed the common peroneal nerve, resulting in the CPNP and exacerbating the neuroparalysis.

**Fig. 1:**
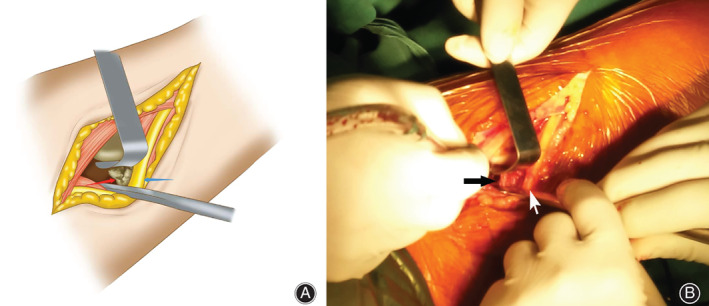
(A) Intraoperative (B) Schematic digram. The red arrow idcicates fabella and the blue arrow indicates common peroneal never.

**Fig. 2:**
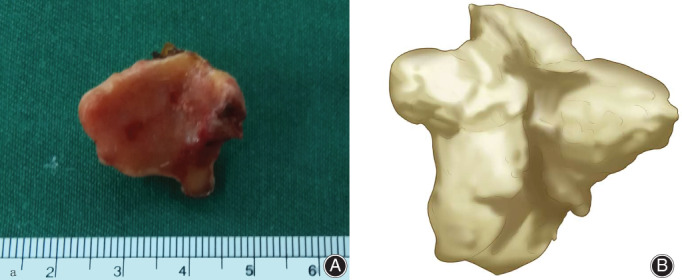
(A) The size of its articular surface of the fabella was approximately 20 × 23mm, and it was 17 mm high. (B) Schematic digram.The removed fabella by surgery.

**Fig. 3:**
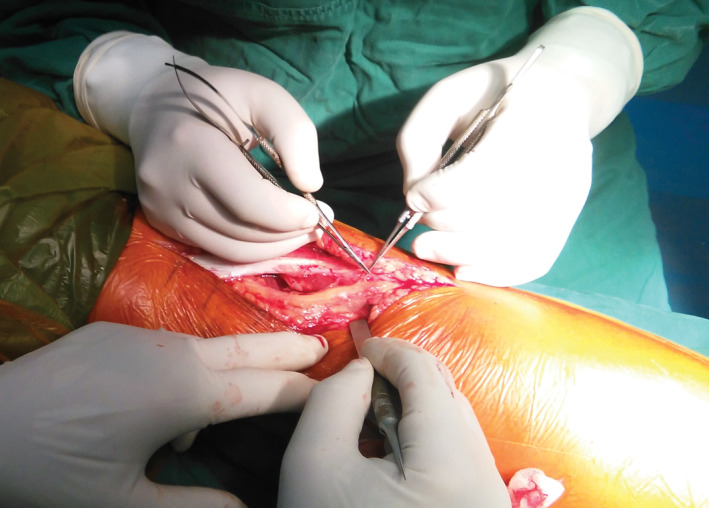
Release of the epineurium of the swollen common peroneal nerve.

Therefore, on the fourth day after TKA, we decided to perform surgical decompression of the nerve. The surgical procedure was performed under general anesthesia and with a tourniquet placed at thigh level. The patient was placed in the lateral decubitus position and a posterolateral oblique incision of the right popliteal fossa was performed, with a length of approximately 10 cm, in line with the course of the nerve. The common peroneal nerve was revealed carefully, and a giant fabella was found in the lateral aspect of the popliteal fossa. The common peroneal nerve was observed to be tightly tented over the giant fabella (Fig. 1). There was a slight swelling and hyperemia in the compression site of the common peroneal nerve. The fabella was carefully removed. The size of its articular surface was approximately 20 × 23 mm and it was 17 mm high (Fig. 2). A nerve release was carefully performed (Fig. 3). Intraoperative tourniquet pressure was 230 mmHg and was used for 20 min.

On the 6th day after the operation of the fabellectomy and nerve decompression, the muscle strength of dorsiflexion of the right ankle and great toe was improved from grade 1/5 to 2/5. At 1 month after the operation, the muscle strength of dorsiflexion of the right ankle, the great toe, and toes was improved to grade 3/5. At 3 months after the surgery, the muscle strength of dorsiflexion of the right ankle, the great toe, and toes was improved to grade 4/5, and the hypoesthesia of the dorsum of the right foot and the inferolateral crural region was significantly reduced. At 8 months postoperatively, the function of the common peroneal nerve fully recovered, the range of motion of the right knee was 0° to 120°, and the patient could walk normally. After 1 year of follow up, the right knee joint function was good, and the common peroneal nerve function was normal.

## Discussion

Common peroneal nerve palsy after TKA is a rare and unforeseen complication. The incidence of previous literature reported was 0.3%–4%^1^, ^2^, ^3^, ^4^. However, Park *et al*. (2013) reported that the actual incidence of CPNP may be underestimated^1^. They believed that some patients with atypical common peroneal nerve impairments were unrecognized and not diagnosed. The risk factors reported for CPNP include: preoperative valgus deformity, preoperative flexion contracture^3^, ^5^, rheumatoid arthritis^12^, prolonged tourniquet time^6^, constrictive dressing, compression from hematomas, postoperative epidural analgesia^10^, higher BMI^1^, previous spinal pathology^9^, ^10^, compression from a pseudotumor^8^, and tibial osteolytic cyst compression^13^. As far as we know, this is the first report of CPNP caused by fabella compression of the common peroneal nerve.

A fabella is a sesamoid bone that is located in the tendon of the lateral head of the gastrocnemius muscle, often directly articulate with the posterior surface of the lateral femoral condyle, and is found bilaterally in most cases^11^. It is visible in approximately 10–30% of the general population; it is, for the most part, asymptomatic and is often overlooked^11^. It is generally believed that its existence is inconsequential to the function or results of the TKA and is often left in place. Rarely, their presence may lead to several pathological conditions, including fabellar pain syndrome^14^, chondromalacia fabellae, and fabella fracture. Following TKA, fabellar impingement upon the posterolateral corner of the polyethylene insert during the knee motion was a source of pain.

The common peroneal nerve, derived from the sciatic nerve, enters the popliteal fossa on the lateral side of the tibial nerve. It runs distally along the medial side of the biceps tendon, crosses posterior to the lateral head of the gastrocnemius muscle, then runs distally between the biceps tendon and the lateral head of the gastrocnemius muscle. The fabella is located in the lateral head of the gastrocnemius tendons.

The CPNP in this case was confirmed during the operation of the giant fabella, which directly oppresses the common peroneal nerve. The possible causes of the fabella oppressing the common peroneal nerve in this case are: the knee flexion contracture was corrected after TKA, which caused the already tensed common peroneal nerve to be oppressed by the fabella as it crosses the fabella; the fabella was giant and located in the front of the common peroneal nerve, and due to surgical stimulation, the surrounding soft tissue was swollen, which caused the common peroneal nerve to be compressed by the fabella; because the patient was thin, there was relatively little soft tissue around the peroneal nerve, which reduced the cushioning effect of the soft tissues, such as fat; and after TKA, the position of the fabella may be changed (e.g. the posterior condylar offset of the femur was increased after the femoral prosthesis was installed, so that the fabella transferred backward to oppress the common peroneal nerve).

To avoid oppression of the common peroneal nerve after TKA, the preoperative radiograph should be carefully scrutinized. If there is a fabella that is larger than normal, thought should be given to its excision during replacement of the knee. Because the fabella is located in the tendon of the lateral head of the gastrocnemius muscle, it can be carefully dissected and removed through the anterior approach prior to component fixation. If fabella oppression of the peroneal nerve is present after TKA, it is better to remove the fabella and decompress the common peroneal nerve through the posterior lateral oblique approach of the popliteal fossa.

Common peroneal nerve palsy due to compression by a giant fabella after TKA is rare. However, surgeons should be aware about such an unusual but potentially devastating complication and treat it promptly and appropriately. Physical examination and appropriate imaging studies are important to make a diagnosis. A careful evaluation of the fabella should be made preoperatively, and if there is an abnormality, the fabella should be removed in TKA to avoid the complication.

## References

[1] Park JH , Restrepo C , Norton R , Mandel S , Sharkey PF , Parvizi J . Common peroneal nerve palsy following total knee arthroplasty prognostic factors and course of recovery. J Arthroplasty, 2013, 28: 1538–1542. 23562462 10.1016/j.arth.2013.02.025

[2] Kaushal SP , Galante JO , McKenna R , *et al*. Complications following total knee replacement. Clin Orthop Relat Res, 1976, 121: 181–187. 991500

[3] Rose HA , Hood RW , Otis JC , Ranawat CS , Insall JN . Peroneal‐nerve palsy following total knee arthroplasty. A review of the Hospital for Special Surgery experience. J Bone Joint Surg Am, 1982, 64: 347–351. 7061551

[4] Schinsky MF , Macaulay W , Parks ML , Kiernan H , Nercessian OA . Nerve injury after primary total knee arthroplasty. J Arthroplasty, 2001, 16: 1048–1054. 11740762 10.1054/arth.2001.26591

[5] Idusuyi OB , Morrey BF . Peroneal nerve palsy after total knee arthroplasty. Assessment of predisposing and prognostic factors. J Bone Joint Surg Am, 1996, 78: 177–184. 8609107 10.2106/00004623-199602000-00003

[6] Horlocker TT , Hebl JR , Gali B , *et al*. Anesthetic, patient, and surgical risk factors for neurologic complications after prolonged total tourniquet time during total knee arthroplasty. Anesth Analg, 2006, 102: 950–955. 16492857 10.1213/01.ane.0000194875.05587.7e

[7] Beller J , Trockel U , Lukoschek M . Peroneal nerve palsy after total knee arthroplasty under continuous epidural anaesthesia. Orthopade, 2008, 37: 475–480. 18415074 10.1007/s00132-008-1257-x

[8] Harvie P , Torres‐Grau J , Beaver RJ . Common peroneal nerve palsy associated with pseudotumour after total knee arthroplasty. The Knee, 2012, 19: 148–150. 21497506 10.1016/j.knee.2011.02.002

[9] Horlocker TT , Cabanela ME , Wedel DJ . Does postoperative epidural analgesia increase the risk of peroneal nerve palsy after total knee arthroplasty? Anesth Analg, 1994, 79: 495–500. 8067554 10.1213/00000539-199409000-00016

[10] Nercessian OA , Ugwonali OF , Park S . Peroneal nerve palsy after total knee arthroplasty. J Arthroplasty, 2005, 20: 1068–1073. 16376265 10.1016/j.arth.2005.02.010

[11] Duncan W , Dahm DL . Clinical anatomy of the Fabella. Clinical Anatomy, 2003, 16: 448–449. 12903068 10.1002/ca.10137

[12] Knutson K , Leden I , Sturfelt G , *et al*. Nerve palsy after knee arthroplasty in patients with rheumatoid arthritis. Scand J Rheumatol, 1983, 12: 201–205. 6623007 10.3109/03009748309098533

[13] Deshmukh AJ , Kuczynski B , Scuderi GR . Delayed peroneal nerve palsy after total knee arthroplasty—a rare complication of tibial osteolysis. The Knee, 2014, 21: 624–627. 24262809 10.1016/j.knee.2013.10.015

[14] Weiner DS , MacNab I . The ‘Fabella syndrome’: an update. J Pediatr Orthop, 1982, 2: 405–408. 6815224

